# Histone Deacetylase 6 Controls Atrial Fibrosis and Remodeling in Postinfarction Mice Through the Modulation of Wnt3a/GSK‐3β Signaling

**DOI:** 10.1096/fj.202500371R

**Published:** 2025-05-16

**Authors:** Shangzhi Shu, Junqiao Fang, Longguo Zhao, Jiatong Han, Meiping Zhang, Chaoqun Huang, Xian Wu Cheng, Shuyan Li

**Affiliations:** ^1^ Department of Cardiovascular Disease The First Hospital of Jilin University Changchun Jilin China; ^2^ Department of Cardiology and Hypertension, Jilin Provincial Key Laboratory of Stress and Cardiovascular Disease Yanbian University Hospital Yanji Jilin China; ^3^ Department of Cardiology, the Wuxi Fifth People's Hospital The Fifth Affiliated Hospital of Jiangnan University Wuxi Jiangshu China

**Keywords:** atrial remodeling, fibrosis, mitochondria, myocardial infarction, oxidative stress

## Abstract

Myocardial infarction (MI)‐induced hemodynamic disorder often causes atrial structural and electrophysiological remodeling. Given that histone deacetylase 6 (HDAC6) plays important roles in pathobiology, we investigated the molecular mechanism underlying MI‐induced atrial remodeling in mice, with a special focus on HDAC6‐mediated Wnt3a/GSK3β signaling activation. We observed an upregulation of HDAC6 expression in the left atria of mice at 2 weeks post‐MI, accompanied by atrial enlargement, increased atrial fibrosis and inflammation, myocyte hypertrophy, impaired mitochondrial biogenesis, elevated levels of Wnt3a, GSK3β, and β‐catenin protein, and reduced gap junction CX43 expression; these alterations were reversed by HDAC6 deletion. This atrialoprotective effect was mimicked by HDAC6 inhibition with the HDAC6 inhibitor tubastatin A (TubA). In HL1 mouse atrial myocytes, HDAC6 silencing (or overexpression) reduced (increased) the Wnt3a and p‐GSK3β protein levels, providing evidence and a mechanistic explanation of HDAC6‐mediated Wnt3a/GSK3β signaling activation in mitochondrial oxidative stress production and cell pyroptosis. After HDAC6 formed a complex with GSK3β and translocated into the mitochondria, GSK3β competitively bound with TFAM to mtDNA, thereby affecting mitochondrial function and ROS generation. The SGLT2 inhibitor dapagliflozin exhibited efficacy that was comparable to that of TubA by inhibiting HDAC6 signaling in mice. These results indicate an essential role of HDAC6 in atrial remodeling in response to post‐MI stress, possibly via the modulation of Wnt3a/GSK3β‐mediated mitochondrial oxidative stress production and pyroptosis and matrix protein production, and they suggest a novel therapeutic strategy for the prevention of post‐MI‐related atrial morphological and electrophysiological remodeling by regulating HDAC6 activity.

## Graphical abstract

Elevated levels of HDAC6 activate the Wnt3a/GSK3β signaling pathway, leading to the phosphorylation of GSK3β and its subsequent binding to HDAC6, followed by translocation into the mitochondria. Within the mitochondria, GSK3β interacts with TFAM, competitively reducing TFAM's binding to mtDNA. This reduction decreases mtDNA transcription and stability, increases the production of ROS, and promotes fibrosis and inflammation, ultimately contributing to atrial remodeling.

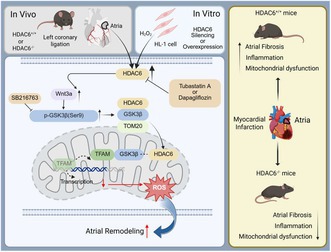

**Abbreviations**
A4C: apical four‐chamber
AR: atrial remodeling
cGAS: cyclic GMP‐AMP synthase
CX43: connexin 43
Dap: dapagliflozin
DCFH‐DA: 2,7‐dichlorodi‐hydrofluorescein diacetate
DHE: dihydroethidium
GSK3β: glycogen synthase kinase 3 beta
HDAC6: histone deacetylase 6
HFpEF: heart failure with preserved ejection fraction
IL: interleukin
LA: left atrium
LAD: left anterior descending coronary
MCP1: monocyte chemoattractant protein 1
MI: myocardial infarction
NLRP3: NOD‐, LRR‐ and pyrin domain‐containing protein 3
OPA1: optic atrophy 1
OPN: osteopontin
P‐T‐GSK3β: phosphorylated or total glycogen synthase kinase 3 beta
PGC1α: peroxisome proliferator‐activated receptor gamma coactivator 1‐alpha
PLAX: parasternal long‐axis view
ROS: reactive oxygen species
SGLT2: sodium‐glucose cotransporter 2
TFAM: mitochondrial transcription factor A
TL: tibia length
TubA: tubastatin A
WGA: wheat germ agglutinin

## 1 Introduction

Atrial arrhythmias (e.g., atrial premature beats, atrial tachycardia, atrial flutter, and atrial fibrillation) are one of the most common types of arrhythmia that can occur after a myocardial infarction (MI) [1]. Clinical and laboratory studies indicate that hemodynamic disorders often cause atrial structural and electrophysiological remodeling after an MI, [2] and serious atrial arrhythmia has been shown to result in atrial remodeling (AR) and dysfunction, providing a possible explanation for the progressive nature of this type of arrhythmia [3, 4]. AR is known to involve complicated processes that are closely linked to harmful changes in inflammation, immunity, oxidative stress, and intracellular signaling pathways [5, 6, 7]. Although the pathophysiological mechanism underlying the genesis of AR has been the focus of many studies, the mechanism remains only partially understood.

An altered oxidative–redox balance has been implicated in the pathogenesis of AR and atrial arrhythmia, and antioxidant drugs have exerted a beneficial effect on both AR and atrial arrhythmia [7, 8]. The mitochondrion is one of the major cellular organelles that are a source of reactive oxygen species (ROS) in the initiation and progression of AR [3]. Mitochondrial injury and dysfunction can increase the production of ROS, leading to adverse atrial structural and electrophysiological remodeling [9]. Inflammation was also observed to lead to cardiomyocyte mitochondrial injury by promoting the depolarization of cardiomyocytes and production of ROS during the progression of atrial fibrillation [10]. Two investigations indicated that glycogen synthase kinase 3 beta (GSK3β) activity is involved in several cellular dysfunctions, [11, 12] and GSK3β/β‐catenin signal activation has been shown to (i) stimulate human atrial fibroblast proliferation and the production of fibronectin and collagen type I and (ii) suppress autophagy [13]. Another study demonstrated that in a murine model of MI, the deletion in cardiac fibroblasts exacerbated fibrosis and left ventricular dysfunction by enhancing TGF‐β1/SMAD‐3 signaling, [14] thus indicating that GSK3β might be a promising molecular target for the management of atrial fibrosis and remodeling.

Histone deacetylase 6 (HDAC6) is a IIb class HDAC that is located primarily in the cytoplasm, where it has critical roles in the regulation of protein degradation, the formation of the cellular microtubule network, and intracellular transport. HDAC6 activation can increase the incidence of atrial fibrillation by disturbing α‐tubulin proteostasis and the homeostasis of cellular microtubule structures [15]. The involvement of HDAC6 was also observed in the assembly of the intracellular NLRP3 inflammasome in macrophages, thereby activating an inflammation response in vitro and in vivo [16]. Inflammation has been confirmed to trigger AR and to promote atrial arrhythmia [17].

HDAC6 was reported to function as a sarcomeric protein deacetylase to modulate cardiac myofibril stiffness and diastolic function in response to aging and hypertension [18]. A 2024 study using mice with heart failure with a preserved ejection fraction (HFpEF) demonstrated that HDAC6 inhibition involves multiple heart failure (HF) mechanisms including hypertrophy, fibrosis, the mitochondrial respiration capacity, and systemic inflammation and metabolism, highlighting the multimodal role of HDAC6 [19]. However, the function of HDAC6 in mitochondrial oxidative stress production and dysfunction during the AR that occurs post‐MI remains largely unclear.

In this study, we created a murine model of MI; the MI was induced by ligation of the left anterior descending (LAD) coronary artery to induce the AR observed in MI patients. We used this model to investigate the impact of inhibiting HDAC6 (i) with a specific inhibitor, tubastatin A (TubA), and (ii) by genetic deletion of HDAC6 on post‐MI AR and the underlying mechanisms. Our findings support a direct role of HDAC6 in MI‐related AR pathophysiology within the heart, indicating that HDAC6 could be a promising molecular target for the management of post‐MI AR. Our results also indicate that the efficacy of TubA is comparable to that of a sodium‐glucose cotransporter 2 (SGLT2) inhibitor, dapagliflozin (Dap).

## 2 Materials and Methods

### 2.1 Experimental Animals

Male HDAC6^+/+^ C57BL/6 mice were provided by Yanbian University Animal Center (Yanbian, China), and male HDAC6^−/−^ mice were purchased from Shanghai Biomodel Organism Science & Technology Development Co. (Shanghai, China; protocol 2022‐W5‐1109). All animals were raised under specific‐pathogen‐free (SPF)‐level conditions with controlled 12‐h light/dark cycles in a humidity and temperature‐controlled environment. The breeding and genotyping of the HDAC6^−/−^ mice were performed by trained research staff. HDAC6 gene knockout was confirmed by agarose gel electrophoresis (Figure S1A). Western blot was applied to evaluate the levels of the acetyl‐α‐tubulin protein (Figure S1B). The animal experimental protocols were reviewed and approved by the Institutional Animal Care and Use Committee at Yanbian University (protocol YD20211128016).

### 2.2 Mouse Model of MI

The mouse MI model was created as described [20]. In brief, 8‐week‐old mice were anesthetized with a 2% isoflurane inhalation oxygen mixture without intubation. An approx. 1‐cm skin incision was made over the left chest, and the 4th intercostal space was identified. A small hole was made at this site, and the heart was gently popped out. A suture (8–0) was placed 2 mm below the origin of the proximal LAD. Successful ligation was defined as the whitening of the left ventricle. The heart was then immediately placed back into the intrathoracic space, air was evacuated, and the chest was closed. The sham mice underwent the same procedure but did not undergo the ligation.

Mice with either genotype were randomly divided into the following six groups: HDAC6^+/+^ mice with the sham operation group (HDAC6^+/+^‐Sham), HDAC6^−/−^ mice with the sham operation (HDAC6^−/−^‐Sham), HDAC6^+/+^ mice that underwent LAD ligation (HDAC6^+/+^‐MI), HDAC6^−/−^ mice that underwent LAD ligation (HDAC6^−/−^‐MI), HDAC6^+/+^ mice that underwent LAD ligation and TubA treatment (MI‐TubA), and HDAC6^+/+^ mice that underwent LAD ligation and treatment with Dap (MI‐Dap).

### 2.3 Tissue Sampling

After they underwent a hemodynamic analysis by echocardiography at the indicated timepoints before and after the MI and sham surgeries (Figure S1C), the mice were anesthetized by an intraperitoneal injection of chloral hydrate (0.1 mL/10 g), and blood samples were isolated from the left ventricles. Following perfusion with 4% phosphate‐buffered saline (PBS) at the physical pressure, the whole heart and left atria were successively separated and weighed. The atrial weight and tibia length (TL) were then measured.

For the biological analyses, the left atrial (LA) tissues were kept in RNAlater solution (Cat.no. AM7020, Invitrogen, Carlsbad, CA) (for the targeted gene expression assay) or stored at −80°C (for the western blotting assay). For the histological and immunostaining analysis, after the LA tissue samples were immersed in fixative at 4°C, they were embedded in optimal cutting temperature (OCT) compound (Sakura Fine‐technical, Tokyo) and stored at −20°C.

### 2.4 Echocardiography and Electrocardiography

Echocardiography and electrocardiography (ECG) examinations were performed 14 days after the MIs. The echocardiography was performed using an 18–38 MHz transducer with a Vevo 2100 system (RRID:SCR_015816, VisualSonics, Toronto, Canada) on mice anesthetized by isoflurane (1%–1.5%) inhalation. Briefly, the LA diameter was measured by using the parasternal long‐axis view (PLAX), and the LA area and E/A ratio were measured using the apical four‐chamber (A4C) view. The LA area/TL was also calculated. ECG data were collected using the PowerLab system (RRID:SCR_018833) and analyzed with LabChart software (RRID:SCR_017551, ADInstruments, Colorado Springs, CO, USA) to assess the P‐wave duration and PR interval.

### 2.5 Histological Analysis

After perfusion, the mouse hearts were fixed with 4% paraformaldehyde and dehydrated in 20% sucrose, followed by cryo‐embedding in OCT. Transverse 5‐μm‐thick LA sections were stained with Masson trichrome (Cat.no. G1340, Solarbio, Beijing, China) and wheat germ agglutinin (WGA) (Cat.no. W11261, Invitrogen) per the manufacturers' instructions, and the cross‐sectional area of myocytes was evaluated from cells that were cut transversely and showed both an intact cell membrane and a nucleus; at least 40–60 cells were calculated per specimen, and the average value for each mouse was used for statistical analysis (×200).

To evaluate the extent of the LA interstitial fibrosis, we selected 4–6 fields at random and counted the ratio of the fibrosis area to the total area of the left atrium with the use of ImageJ software (RRID: SCR_003070, ver. v1.54). An Invitrogen EVOS FL Auto2 microscope and EVOS FL Auto Imaging System Software (RRID:SCR_026039, ver. v1.6) were used to record images of the LA interstitial fibrosis.

### 2.6 Electron Microscopy Assay

LA tissue was prefixed with 4% glutaraldehyde and then postfixed with 1% osmium tetroxide. After undergoing dehydration through a series of graded ethanol, the tissue was subsequently embedded in Eponate 12 epoxy resin as described [21]. With the use of an ultramicrotome (EM UC7; Leica Microsystems, Wetzlar, Germany), semi‐thin sections were carefully positioned and then subjected to ultrathin sectioning. These sections were double‐stained with uranyl acetate and lead citrate and ultimately observed and imaged with a transmission electron microscope (FEI Tecnai Spirit; Camcor, Eugene, OR) operating at 100 kV. The quantitation of the mitochondrial numbers and damage was done at a magnification of 15,000 × by counting the corresponding number of pixels using Adobe Photoshop CS5 software. A total of 20 mitochondrial cross‐sections from 4 to 6 sections were calculated and averaged for each mouse, and distribution diagrams were obtained separately for each of the groups.

### 2.7 Cell Culture and Transfection

HL1 mouse atrial myocytes were purchased from Cellverse Bioscience Technology (RRID: CVCL_0303, Shanghai, China). All cell lines were cultured at 37°C in a 5% CO_2_ atmosphere in Dulbecco's modified Eagle's medium (DMEM) (Vivacell Biosciences, Shanghai, China) supplemented with 10% fetal bovine serum (Pricella, Wuhan, China). Small interfering RNA against HDAC6 (siRNA‐HDAC6), HDAC6 plasmid complementary DNA (pcDNA‐HDAC6), and all negative control vectors were constructed by Hanheng Biotechnology Company (Shanghai, China). As shown in Figures 5A, 7A, and 8A, cell transfection was performed using Lipofectamine 3000 reagent (Cat.no. L3000015, Thermo Fisher Scientific, Waltham, MA) according to the manufacturer's instructions.

After transfection, the cells were cultured for an additional 48 h, and the transfection efficiency was assessed in fluorescence microscopy and western blotting assays (Figure S2A,B). To induce a cellular oxidative stress model, we treated the cells with 600 μM H_2_O_2_ (Cat.no. 7722‐84‐1, Merck) for 24 h; the control group was treated with PBS. The GSK3β inhibitor SB216763 (Cat.no. 280744‐09‐4, MCE) dissolved in DMSO was applied to siRNA‐transfected cells at a concentration of 5 μM, and the control group was given an equivalent amount of DMSO.

### 2.8 Quantitative Real‐Time Polymerase Chain Reaction (qPCR)

Total RNA was extracted from tissue using Trizol reagent (Invitrogen). We performed a quantitative real‐time polymerase chain reaction (qRT‐PCR) analysis to determine the mRNA levels of HDAC6, NOD‐, LRR‐ and pyrin domain‐containing protein 3 (NLRP3), osteopontin (OPN), Caspase1, interleukin (IL)‐1β, Wnt3a, β‐catenin, peroxisome proliferator‐activated receptor gamma coactivator 1α (PGC1α), mitochondrial transcription factor A (TFAM), optic atrophy 1 (OPA1) with a QuantiNova SYBR Green‐based qPCR Kit (Cat.no. 208252, Qiagen, Hilden, Germany) and a 7300 Plus RT‐PCR system (RRID:SCR_019335, Thermo Fisher Scientific). The results were normalized to GAPDH expression. All of the primers were synthesized by Thermo Fisher Scientific, and the sequences are listed in Table S1.

### 2.9 Western Blotting

Protein from LA tissue of mice or cultured cells was extracted using radioimmunoprecipitation assay (RIPA) lysis buffer (Solarbio) that was supplemented with 1% Protein Phosphatase Inhibitor and 1% phenylmethylsulfonyl fluoride (PMSF). Protein lysates were separated by sodium dodecyl sulfate‐polyacrylamide gel electrophoresis (SDS‐PAGE) and analyzed by immunoblotting using the following first antibodies: rabbit monoclonal anti‐HDAC6 (Cat.no. 7612), rabbit anti‐phospho‐GSK‐3α/β (Ser21/9) (Cat.no. 9331), rabbit monoclonal anti‐GSK‐3α/β (Cat.no. 5676), rabbit anti‐Wnt3a (Cat.no. 2391), rabbit monoclonal anti‐β‐Catenin (Cat.no. 8480), rabbit monoclonal anti‐NLRP3 (Cat.no. 15101), rabbit monoclonal anti‐caspase 1 (Cat.no. 24232), rabbit monoclonal anti‐GAPDH (Cat.no. 2118), rabbit anti‐TOM20 (Cat.no. 42406; 1:1000 for each, all from Cell Signaling Technology [CST], Beverly, MA), rabbit anti‐alpha‐SMA (1:1000, Cat.no. AF1032, Affinity Biosciences, Cincinnati, OH), rabbit anti‐Collagen I (1:1000, Cat.no. ab260043, Abcam, Cambridge, MA), rabbit polyclonal anti‐Connexin 43 (1:750, Cat.no. 26980‐1‐AP, Proteintech, Wuhan, China), rabbit anti‐cleaved‐caspase 1 (1:1000, Cat.no. AF4005, Affinity), rabbit anti‐IL‐1beta (1:1000, Cat.no. ab283818, Abcam), and rabbit polyclonal anti‐TFAM (1:1000, Cat.no. 22586‐1‐AP, Proteintech). The PVDF membranes (Millipore, Bedford, MA) were incubated with horseradish peroxidase (HRP)‐conjugated secondary antibody (1:5000, Cat.no. 7074, CST). All data were acquired by an Azure 600 ultimate near‐infrared imaging system (RRID:SCR_023780, Azure Biosystems, Dublin, CA) and analyzed with ImageJ software.

### 2.10 Laboratory Analyses

After mouse blood was collected and allowed to settle, it was centrifuged at 4000 rpm to obtain serum. The secretions of IL‐1β and IL‐18 were then measured with an enzyme‐linked immunosorbent assay (ELISA) kit according to the manufacturer's instructions (Cat.no. KE10003, KE00193, Proteintech).

### 2.11 ROS Detection and Mito SOX/Tracker Staining

Reactive oxygen species in mouse atrial tissue sections were detected by staining with 10 μM dihydroethidium (DHE) (Cat.no. 38483–26‐0, Yeasen, Shanghai, China). The intracellular ROS of HL1 cells were measured by staining with 10 μM DCFH‐DA (2,7‐dichlorodi‐hydrofluorescein diacetate, Cat.no. 4091‐99‐0, MCE). Both the slides and sections were stained for 30 min. We used 5 μM Mito SOX (Mitochondrial Superoxide Indicator, Cat.no. 167095–09‐2, Yeasen) to stain mitochondrial superoxide and 200 nM Mito Tracker (Cat.no. 201860–17‐5, Yeasen) to label mitochondria. All samples were visualized by an Invitrogen EVOS FL Auto2 microscope and analyzed with ImageJ software.

### 2.12 Immunofluorescence Staining

Immunofluorescence staining was performed as described [22]. In brief, 5‐μm sections of mice LA tissue were fixed with 4% paraformaldehyde for 15 min. Antigen retrieval was performed using Citrate‐EDTA Antigen Retrieval Solution (Beyotime, Shanghai, China) followed by 0.3% Triton‐X 100 for 15 min. The sections were then blocked with 3% goat serum at room temperature for 1 h and incubated with the primary antibody overnight (mouse anti‐HDAC6, Cat.no. 67250‐1‐Ig, Proteintech; rabbit anti‐CX43, Cat.no. 26980‐1‐AP, Proteintech; mouse anti‐Alpha Actinin, Cat.no. 66895‐1‐Ig, Proteintech; rabbit monoclonal anti‐Galetin 3, Cat.no. ab76245, Abcam). The cells were next fixed with 4% paraformaldehyde for 10 min, treated with 0.3% Triton‐X 100 for 10 min, blocked with 3% goat serum for 30 min, and incubated with the primary antibody overnight (mouse anti‐HDAC6, Cat.no. 67250‐1‐Ig, Proteintech; mouse anti‐GSK3β, Cat.no. 22104‐1‐AP, Proteintech; rabbit anti‐TOM20, Cat.no. 42406, CST). All of the samples were then incubated with Alexa Fluor 488 or 594 fluorophore‐labeled secondary antibody (Cat.no. K1204, K1214, K3306, K3305, APExBIO, Houston, TX) for 1 h. Finally, the nuclei were stained with DAPI. Pyroptotic cells were detected by using a Hoechst 33342/Propidium Iodide (PI) Double Stain Kit (Cat.no. CA1120, Solarbio). All sections and slides were observed and photographed with the Invitrogen EVOS FL Auto2 microscope and analyzed with ImageJ software.

### 2.13 Co‐Immunoprecipitation Assay

Cell lysates from the HL1 cells were collected and lysed in the mixed lysis buffer mentioned above. Protein A/G Magnetic Beads (Cat.no. HY‐K0202, MedChemExpress, Monmouth Junction, NJ) were incubated with 1–2 μg of antibody (rabbit anti‐HDAC6, Cat.no. 7612, CST; rabbit anti‐GSK3β, Cat.no. 9315, CST; rabbit anti‐TFAM, Cat.no. 22586‐1‐AP, Proteintech) or IgG Isotype control antibody (Cat.no. 3900, CST) at 4°C for 1 h and then incubated with cell lysates for 3 h. The beads and matrix were washed following the manufacturer's instructions. The precipitates were washed three times with phosphate‐buffered saline with Tween‐20 (PBST) before the immunoblotting assay.

### 2.14 Using the HDOCK Server for the Prediction of Molecular Docking

The molecular docking method that we used was as described [23]. The protein structures were obtained from the UniProt database (RRID:SCR_002380, https://www.uniprot.org/). The optimal X‐ray crystal structure 6AE3 of GSK3B_MOUSE (Uniprot ID: Q9WV60) was selected as the ligand protein, and the full‐length AlphaFold‐predicted structure of TFAM_MOUSE (Uniprot ID: P40630) was chosen as the receptor protein. Protein–protein docking was performed using the local HDOCKlite v1.1 server (RRID:SCR_024799). An interaction analysis was performed using the PDBePISA server (RRID:SCR_015749) [24].

### 2.15 Statistical Analyses

We used GraphPad Prism software (RRID:SCR_002798, ver. 9.5, La Jolla, CA) to analyze the results. All data are presented as the mean ± standard deviation. The unpaired Student's *t*‐test was performed to compare the two‐sample comparisons. The differences between multiple groups were measured by a one‐way analysis of variance (ANOVA) with Tukey's post hoc test. After we determined the status of the data distribution, the data were subjected to the statistical analysis. If the homogeneity of variance assumption was violated, the nonparametric Kruskal–Wallis test was used instead of Pearson's chi‐square or Fisher's exact test. Morphological and histological characteristics were evaluated by two observers in a blind manner, and the values they obtained were averaged. Probability (*p*)‐values < 0.05 were considered significant.

## 3 Results

### 3.1 The HDAC6 Expression Was Increased in LA Tissues of Post‐MI Mice

**FIGURE 1:**
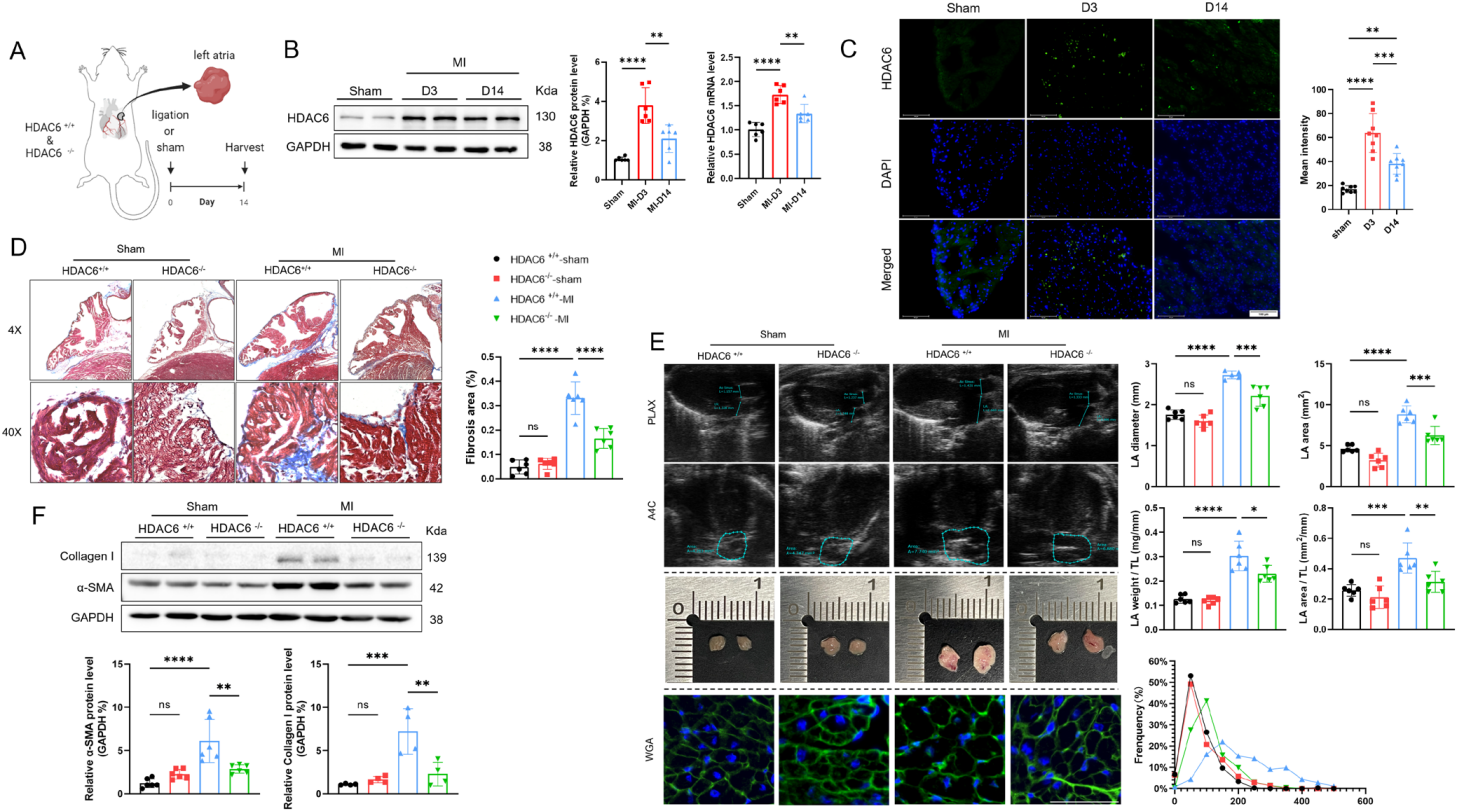
Increased expression of HDAC6 in the left atrial (LA) post‐myocardial infarction (MI) contributes to atrial fibrosis and enlargement, and this is alleviated by HDAC6 deficiency. (A) Illustration of the experimental protocol. (B, C) HDAC6 protein, mRNA levels, and immunofluorescence (scale bar: 100 μm) were evaluated in the sham group and at 3 and 14 days post‐MI. (D, F) Masson's staining of the LA at 14 days post‐MI, along with the western blot of fibrosis‐related proteins (α‐SMA and collagen I). The results of the quantitative analysis of the fibrosis area and protein levels are shown. (E) Representative echocardiography, gross specimens, and wheat germ agglutinin (WGA) staining (scale bar: 50 μm) of the LA at 14 days post‐MI, performed to evaluate LA enlargement and cardiomyocyte size. The LA diameter, LA area, LA weight/TL (tibia length), LA area/TL, and the frequency distribution of cell size were evaluated separately. *p < 0.05, **p < 0.01, ***p < 0.001, ****p < 0.0001. A4C, apical four‐chamber view; DAPI, 4′: 6‐diamidino‐2‐phenylindole; PLAX, parasternal long‐axis view.

To study the impact of MI on the HDAC6 expression in the mouse LA, HDAC6^+/+^ mice that had undergone a sham operation or LAD ligation surgery were subjected to echocardiography and biological analyses at the time points indicated in Figure 1A and Figure S1C. We observed that on the post‐MI day 14, the MI mice showed harmful changes in the AR‐related LA diameter and LA area (Figure S3A). The qPCR and western blotting data revealed that the HDAC6 mRNA and protein levels of the LA tissues were markedly increased during the follow‐up period and reached respective 1.7‐ and 3.7‐fold peaks at post‐MI induction day 3 (Figure 1B). The quantitative immunostaining yielded the same conclusion (Figure 1C), indicating that HDAC6 might be involved in the MI‐related AR in mice under our experimental conditions.

### 3.2 The Deletion of HDAC6 Alleviated LA Fibrosis and Remodeling

To explore the role of HDAC6 in the AR of the post‐MI mice, we subjected HDAC6^+/+^ and HDAC6^−/−^ mice that had undergone the sham or MI surgeries to echocardiography, electrocardiography, and sampling on post‐MI Day 14 (Figure S1C). The results demonstrated that HDAC6 deletion significantly reduced the atrial interstitial fibrosis area, LA weight/TL ratio, LA area/TL ratio, LA diameter and LA area, and cardiomyocyte size compared to the HDAC6^+/+^‐MI mice (Figure 1D,E). As anticipated, the MI surgery resulted in increased levels of type I collagen and α‐SMA proteins in the LA, and these alterations were rectified by HDAC6 deletion (Figure 1F). The ECG of post‐MI mice exhibited a prolonged a P wave and PR interval in the HDAC6^+/+^ MI group, and both alterations were reversed by HDAC6 deletion (Figure S3B). HDAC6 thus appears to participate in atrial structural and electrophysiological remodeling. At the baseline, there were no significant differences between the two mouse genotypes (i.e., HDAC6^−/−^ and HDAC6^+/+^ C57BL/6) in the LA weight/TL ratio, LA size, atrial cardiomyocyte size, fibrosis, or the investigated protein levels (Figure 1D–F).

### 3.3 HDAC6 Deletion Lowered the LA Wnt3a‐GSK3β Signaling Activation and Improved the CX43 Expression and Mitochondrial Biogenesis in Post‐MI Mice

**FIGURE 2:**
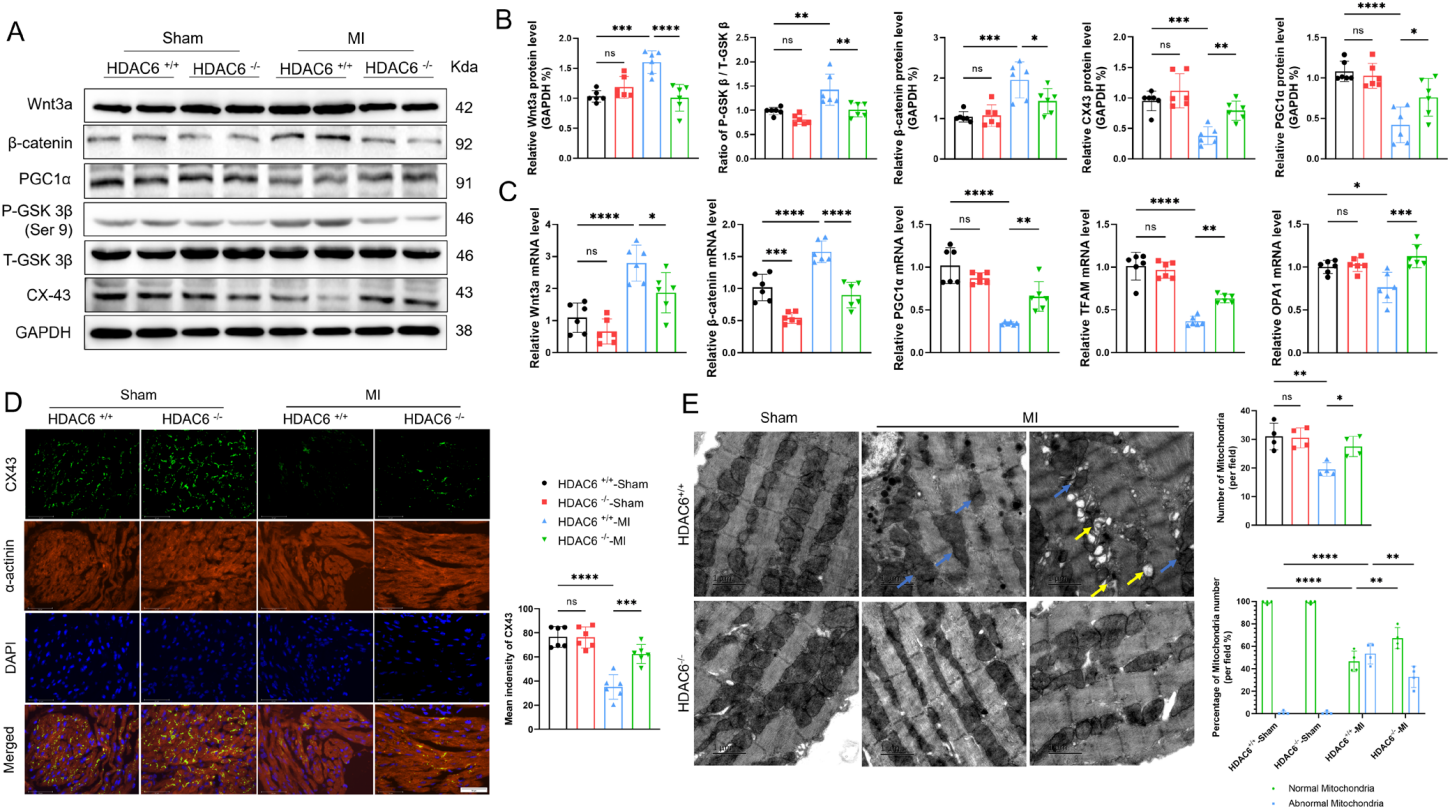
HDAC6 deficiency affects the Wnt/GSK3β signal pathway, inhibits CX43 downregulation, and restores mitochondrial function. (A, B) The protein level of wnt3a, β‐catenin, PGC1α, CX43, P‐GSK3β, and T‐GSK3β by western blot. The results of the quantitative analysis of Wnt3a, β‐catenin, PGC1α, the CX43 level, and the ratio of P‐GSK3β/T‐GSK3β are shown. (C) The relative mRNA levels of Wnt3a, β‐catenin, PGC1α, TFAM, and OPA1. (D) Immunofluorescence of CX43 (*green*), α‐actinin (*red*), and DAPI (*blue*) (scale bar: 50 μm). The results of the quantitative analysis of CX43 fluorescence intensity are shown. (E) Transmission electron microscopy images of mitochondria (scale bar: 1 μm). *p < 0.05, **p < 0.01, ***p < 0.001, ****p < 0.0001. CX43, connexin 43; P‐T‐GSK3β, phosphorylated or total glycogen synthase kinase 3 beta; PGC1α, peroxisome proliferator‐activated receptor gamma coactivator 1‐alpha; TFAM, mitochondrial transcription factor A.

To explore whether the Wnt/GSK3β signaling pathway is involved in LA remodeling after MI, we conducted western blot analyses of the related intracellular signal proteins. As shown in Figure 2A, on post‐MI day 14, the immunoblot analysis using equal amounts of protein from each sample showed 1.3‐ to 1.8‐fold increases of Wnt3a, β‐catenin, and p‐GSK3β (at Ser9) in the LA tissues of the HDAC6^+/+^‐MI mice. These changes were reversed by 24%–38%, respectively, in the LA tissues of the HDAC6^−/−^‐MI mice. The qPCR yielded the same conclusions regarding the Wnt3a and β‐catenin gene expressions (Figure 2C), indicating that HDAC6 can modulate left atrial Wnt3a‐GSK3β signaling activation in post‐MI mice. Conversely, the LA of the HDAC6^+/+^‐MI mice showed reduced levels of p‐GSK3β (at Tyr216) protein, which was increased in the HDAC6^−/−^‐MI mice (Figure S4C).

Abnormalities in cardiomyocyte gap junctions are closely associated with the LA structural and electrical remodeling in humans and rats with atrial fibrillation [25]. Immunofluorescent double staining detected a marked reduction in the CX43 protein expression in the LA tissues of post‐MI HDAC6^+/+^ mice, and this alteration was reversed by HDAC6 deletion (Figure 2D). This was consistent with the western blotting analysis result, i.e., HDAC6^−/−^ prevented this reduction in post‐MI mice (Figure 2A,B). PGC1α and TFAM have been shown to modulate mitochondrial gene expression and biogenesis [26, 27]. HDAC6 deficiency also elevated the levels of PGC1α, TFAM, and OPA1 genes and/or proteins in the LA tissues of post‐MI mice (Figure 2A–C). As depicted in Figure 2E, the transmission electron microscopy findings combined with the quantitative data revealed that the LA tissues of post‐MI HDAC6^+/+^ mice had markedly disorganized myofibril arrangement, mitochondrial membranes and cristae structures, vacuole formation, and expanded sarcoplasmic reticulum; these phenomena were ameliorated by HDAC6 deletion (Figure 2E). Collectively, these data suggested that the lack of HDAC6 in particular might prevent the atrial microstructural changes of the mitochondrial and cardiomyocyte gap junction observed in post‐MI mice.

### 3.4 HDAC6 Deficiency Reduces Atrial Oxidative Stress and Inflammation in Post‐MI Mice

**FIGURE 3:**
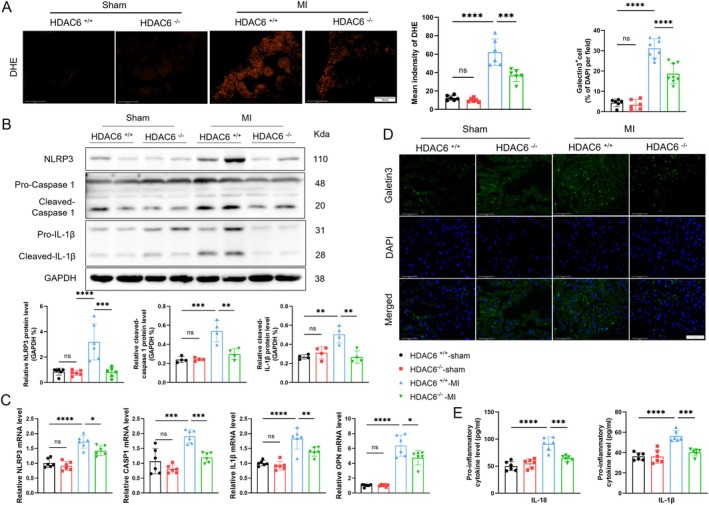
HDAC6 deficiency alleviates oxidative stress and inflammation in the LA post‐MI. (A) DHE staining of LA tissue 3 days post‐MI (scale bar: 100 μm). The results of the quantitative analysis of fluorescence intensity are shown. (B) Western blot of NLRP3, pro‐caspase1, cleaved‐caspase1, pro‐IL1β, cleaved‐IL1β, and CX43. The results of the quantitative analysis of NLRP3, cleaved‐caspase1, cleaved‐IL1β, and CX43 are shown. (C) The relative mRNA levels of NLRP3, CASP1, IL1β and OPN. (D) Representative immunofluorescence staining of galectin 3 (*green*) and DAPI (*blue*). The results of the quantitative analysis of galectin3‐positive cells are shown. (E) The ELISA assay measurements of the serum IL‐1β and IL‐18 concentrations. *p < 0.05, **p < 0.01, ***p < 0.001, ****p < 0.0001. DHE, dihydroethidium; OPN, osteopontin.

Oxidative stress and inflammation have been shown to be associated with the development of post‐MI AR, thus significantly contributing to atrial arrhythmia [28]. In the present study, the quantitative data of DHE staining revealed a significant increase in ROS levels in the HDAC6^+/+^‐MI group, and these levels were significantly reduced in the HDAC6^−/−^‐MI group (Figure 3A). Inflammation mediated by the NLRP3 inflammasome is particularly critical in this process [17]. We observed that in the HDAC6^+/+^‐MI group, NLRP3 was increased in the LA tissues of mice at 14 days post‐MI, accompanied by elevated levels of cleaved‐caspase1 and cleaved‐IL1β proteins (Figure 3B). HDAC6 deficiency alleviated these elevations, showing similar changes (i.e., NLRP3, caspase1, and IL‐1β) at the mRNA level (Figure 3C). HDAC6 deletion also led to the attenuation of an MI‐induced atrial inflammatory response, including reduced galectin3^+^ macrophage infiltration (Figure 3D) and decreased levels of plasma IL‐1β and IL‐18 and atrial OPN (Figure 3C,E) evaluated by the immunofluorescence, ELISA, and qPCR. These results indicate that HDAC6^−/−^‐mediated reductions of inflammation and oxidative stress production might contribute to beneficial atrial effects in post‐MI mice under our experimental conditions.

### 3.5 Tubastatin A and Dapagliflozin Treatment Were Beneficial for AR in the Post‐MI Mouse LA Tissue

**FIGURE 4:**
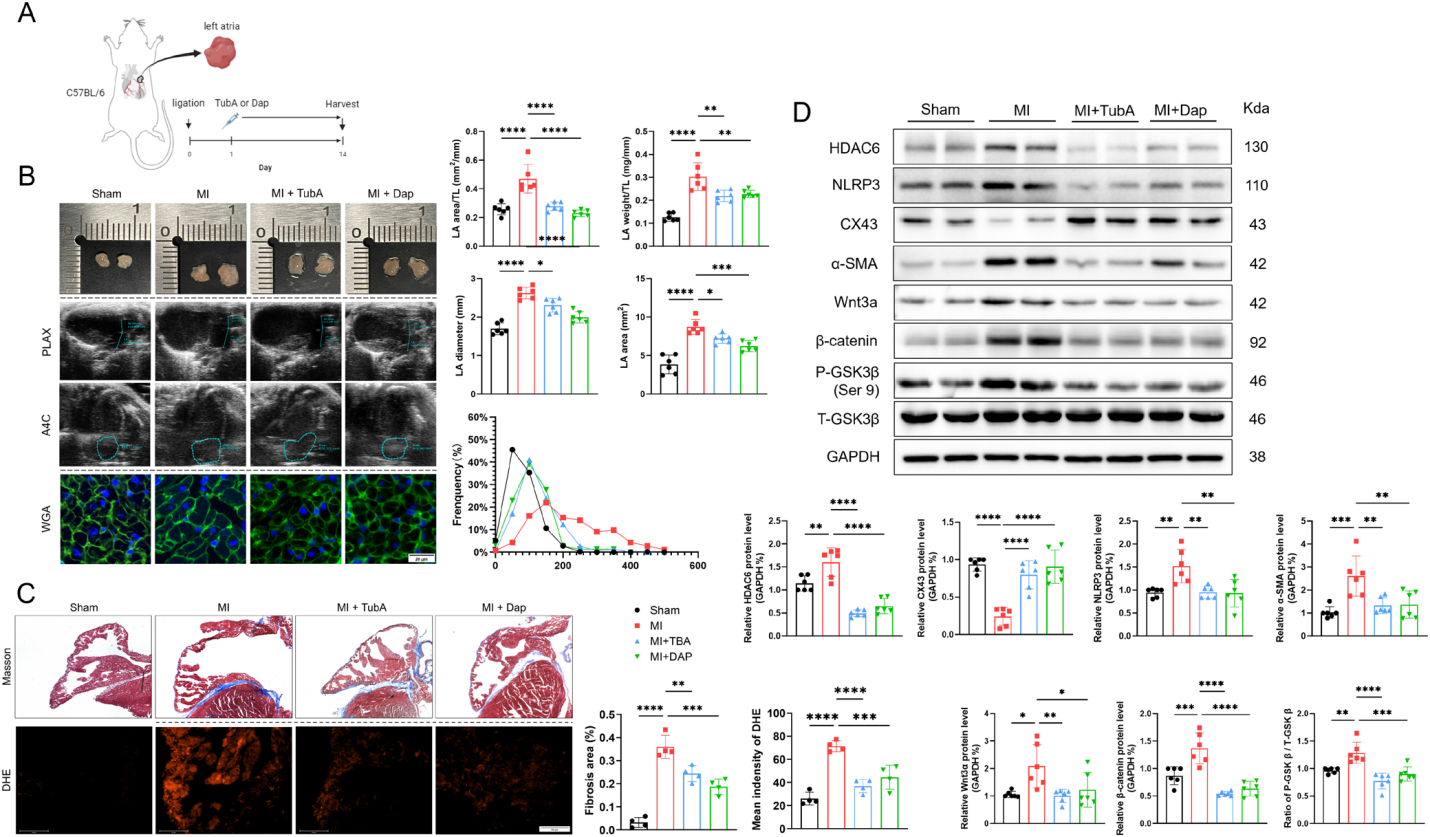
Tubastatin A and dapagliflozin can inhibit HDAC6 and alleviate atrial remodeling post‐MI. (A) Illustration of the experimental protocol. (B) Representative echocardiography, gross specimens, and WGA staining results (scale bar: 50 μm) of atrial tissue after 14 days of drug treatment. The LA diameter, LA area, LA weight/TL, LA area/TL, and the frequency distribution of cell size were evaluated separately. (C) Masson staining of the LA after 14 days of drug treatment and the results of the quantitative analysis of the fibrosis area. DHE staining of LA after 3 days of drug treatment and the quantitative analysis results concerning fluorescence intensity. (D) The western blot and quantitative analysis of HDAC6, NLRP3, CX43, α‐SMA, Wnt3a, β‐catenin, P‐GSK3β, and T‐GSK3β. *p < 0.05, **p < 0.01, ***p < 0.001, ****p < 0.0001.

Tubastatin A (TubA, an HDAC6 inhibitor) and dapagliflozin (Dap, an SGLT2 inhibitor) have been shown to exert a cardiovasculoprotective effect on hypoxia and non‐hypoxia‐induced cardiovascular injuries in patients and animal models [29, 30]. One study has suggested that SGLT2 inhibitors may serve as targeted inhibitors of HDAC6 [31]. To investigate whether these drugs could have a beneficial effect on MI‐induced AR and inhibit HDAC6, we treated HDAC6^+/+^ mice that had undergone the MI surgery with or without TubA (15 mg/kg/day) or Dap (10 mg/kg/day) from day 1 to day 14 and then subjected them to the related analyses (Figure 4A). The TubA treatment markedly improved the MI‐related LA parameter changes including the atrial interstitial fibrosis area, weight/TL ratio, LA diameter, LA area, and LA area/TL ratio and cardiomyocyte size as well as the oxidative stress production compared to the HDAC6^+/+^‐MI alone mice (Figure 4B,C). HDAC inhibition also mitigated the harmful molecular changes including HDAC6/Wnt3a signaling (HDAC6, Wnt3a, β‐catenin, p‐GSK‐3β at Ser 9)‐, inflammation (NLRP3)‐, cardiomyocyte gap junction (CX43)‐, and fibrotic (α‐SMA)‐related proteins in the LA tissues of HDAC6^+/+^‐MI mice (Figure 4D). In addition, Dap as well as TubA exhibited an inhibitory effect on HDAC6 expression, which was further validated by the elevation of acetylation of α‐tubulin (Figure S4B). In HL‐1 cells, both drugs exhibited an inhibitory effect on HDAC6 expression (Figure S4C). We observed comparable efficacy between TubA and Dap on the AR in HDAC6^+/+^‐MI mice. These observations supported our hypothesis regarding HDAC6 inhibition‐mediated atrial protective actions in HDAC6^+/+^‐MI mice under our experimental conditions.

### 3.6 HDAC6 Silencing Alleviated Oxidative‐Induced Wnt3a/GSK3β Activity, Pyroptosis, and CX43 Downregulation in HL1 Cells

**FIGURE 5:**
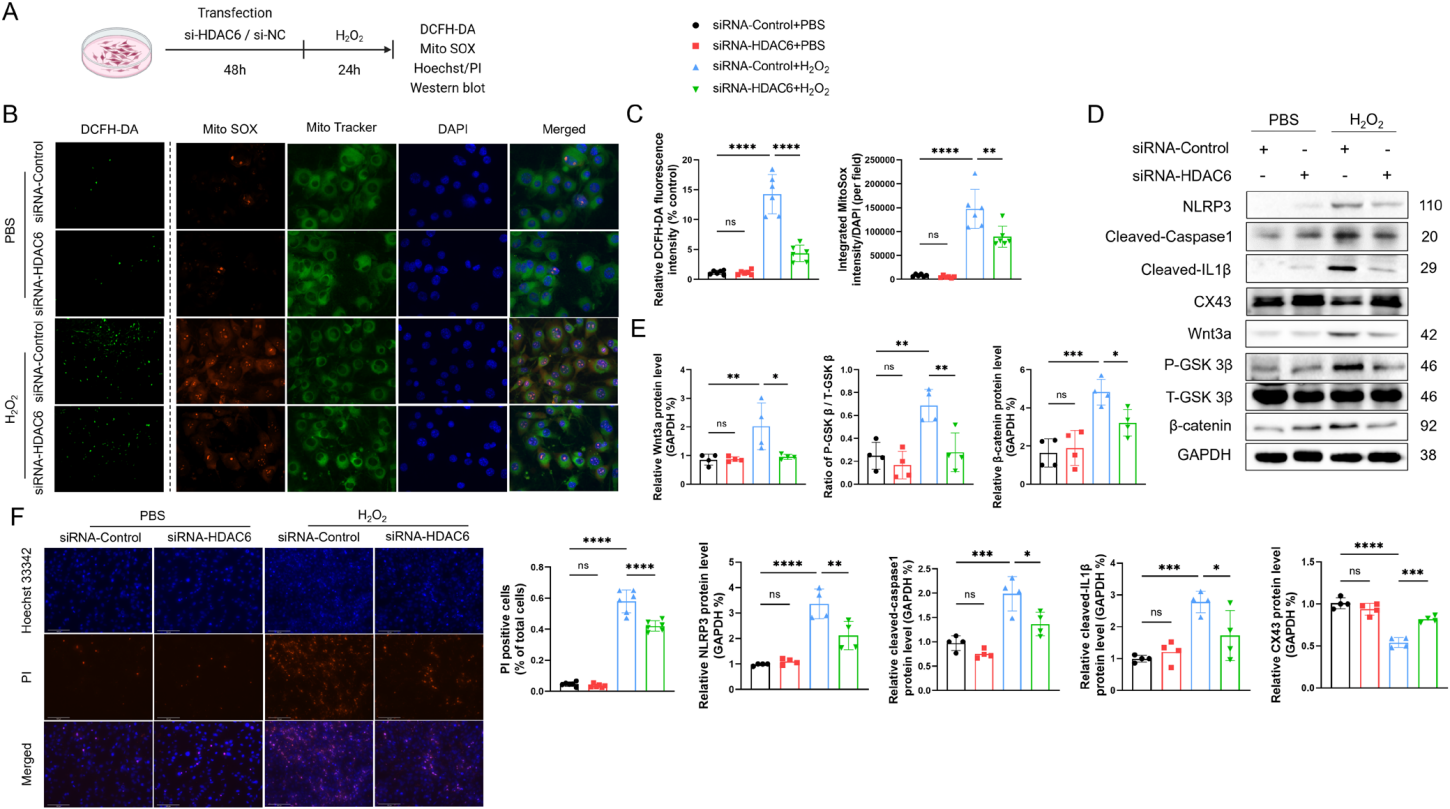
HDAC6 knockout alleviates H_2_O_2_‐induced pyroptosis, oxidative stress, and CX43 downregulation in HL1 cells. (A) Illustration of the transfection and treatment experimental protocol. (B, C) DCFH‐DA (scale bar: 100 μm) and Mito SOX (*red*)/Mito Tracker (*green*) (scale bar: 50 μm) staining of cells after siRNA transfection and H_2_O_2_ treatment. The results of the quantitative analysis of fluorescence intensity are presented. (D, E) Western blot and quantitative analysis of NLRP3, cleaved‐caspase1, cleaved‐IL1β, CX43, Wnt3a, P‐GSK3β, T‐GSK3β and β‐catenin. (F) Hoechst/PI staining of cells in each group. The results of the quantitative analysis of PI‐positive cells are shown. *p < 0.05, **p < 0.01, ***p < 0.001, ****p < 0.0001. DCFH‐DA, 2′,7′‐dichlorodihydrofluorescein diacetate; MitoSOX, mitochondrial superoxide indicator; PI, propidium iodide.

We first evaluated the efficacy of HDAC6 silencing by immunofluorescence and western blotting assays (Figure S2A). We observed an H_2_O_2_‐mediated dose‐dependent response to the HDAC6 induction of HL1 cells, with the highest response at 600 μM (Figure S2C). We divided HL1 cells into four groups: siRNA‐Control+PBS, siRNA‐HDAC6 + PBS, siRNA‐Control+H_2_O_2_, and siRNA‐HDAC6 + H_2_O_2_. The cells were transfected with siRNA‐HDAC6 or a transfected negative control (NC), respectively, followed by H_2_O_2_ treatment (Figure 5A). As anticipated, the H_2_O_2_ treatment induced a significant increase in ROS production, confirmed by a stronger DCFH‐DA signal (Figure 5B,C). Mito Tracker/Mito SOX is a probe that has often been used to evaluate the changes in transmembrane potentials as well as ROS generation [32] We observed a markedly elevated staining signal in H_2_O_2_‐treated HL1 cells; these changes were alleviated following the knockdown of HDAC6 (Figure 5B,C). HDAC6 silencing also ameliorated the harmful changes in the levels of pyroptosis‐related proteins including NLRP3, cleaved‐caspase1, and cleaved‐IL1β proteins as well as the cardiomyocyte cap‐junction‐related CX43 protein (Figure 5D,E). Also, the H_2_O_2_ treatment resulted in increased levels of Wnt3a, p‐GSK3β, and β‐catenin, and these changes were markedly recovered by HDAC6 silencing in the cells (Figure 5D,E). Pyroptosis is characterized by cell swelling, the release of pro‐inflammatory intracellular components, and pore formation on the cell membrane, leading to positive staining with propidium iodide (PI). The Hoechst/PI staining performed herein showed that the H_2_O_2_ treatment resulted in an increase in the PI^+^ cells, and this increase was significantly alleviated by HDAC6 knockdown (Figure 5F). These results together with those of the in vivo experiments indicate that the absence of HDAC6 can alleviate atrial cardiomyocyte pyroptosis and gap‐junction formation in post‐MI mice.

### 3.7 HDAC6 Bound to GSK‐3β Translocated Into the Mitochondria in HL1 Cells in Response to H_2_O_2_

**FIGURE 6:**
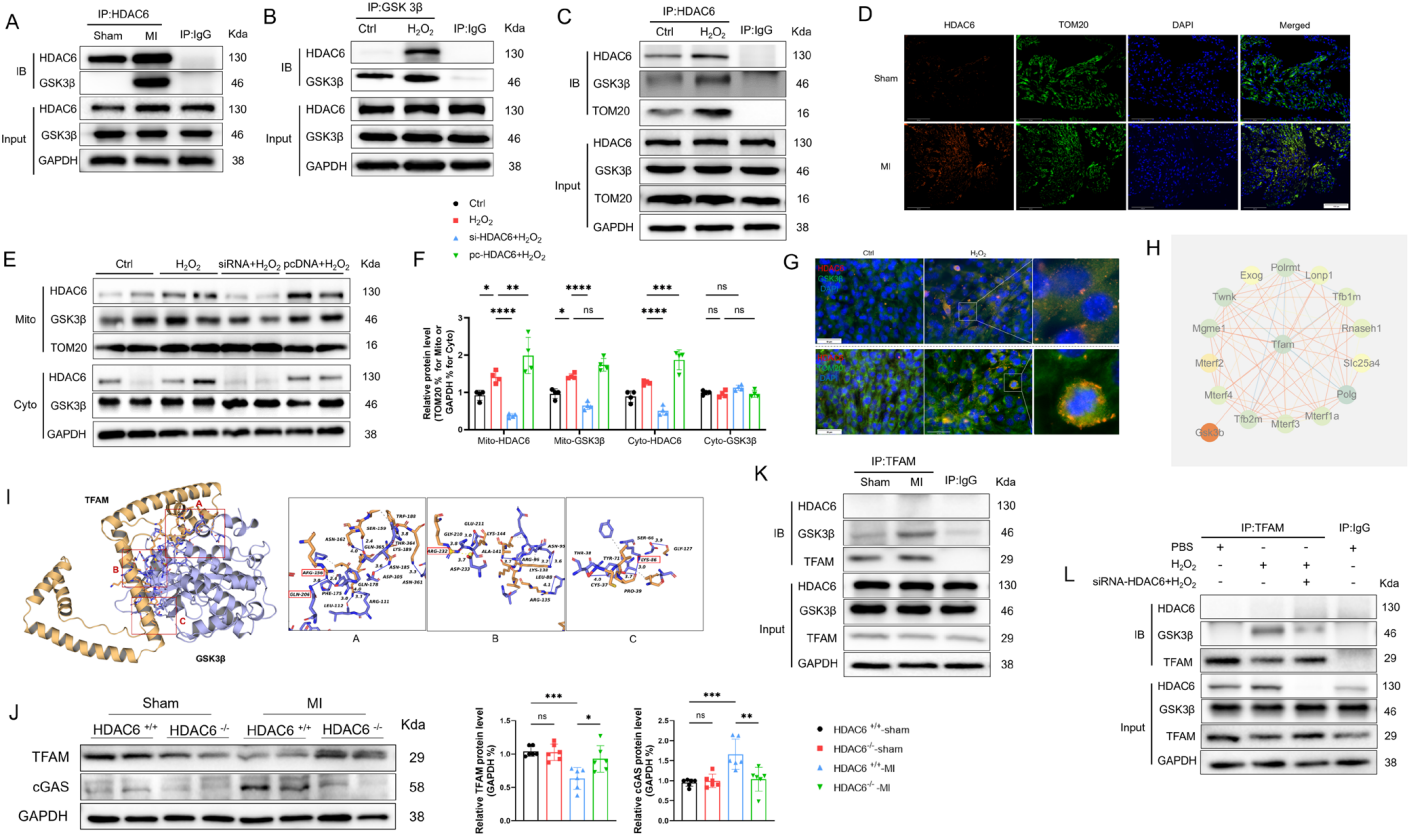
HDAC6 binds to GSK3β and translocates to the mitochondria in H_2_O_2_‐induced HL1 cells and atria post‐MI. (A) Co‐immunoprecipitation using HDAC6 antibody in the LA after MI. (B) Co‐immunoprecipitation using GSK3β antibody in cells with or without H_2_O_2_ induction. (C) Co‐immunoprecipitation using HDAC6 antibody or IgG Isotype antibody. Immunoblotting was performed for GSK3β, HDAC6, and TOM20. (D) Immunofluorescence of TOM20 (*green*), HDAC6 (*red*), and DAPI (*blue*) (scale bar: 100 μm). (E, F) Western blotting was conducted separately for HDAC6 and GSK3β in mitochondrial and cytoplasmic fractions, using TOM20 as the mitochondrial reference marker and GAPDH as the cytoplasmic reference marker. The results of the quantitative analysis of HDAC6 and GSK3β are shown. (G) Cell immunofluorescence staining, from top to bottom: HDAC6 (*red*) and GSK3β (*green*), and HDAC6 (*red*) and TOM20 (*green*), with all nuclei stained using DAPI (*blue*) (scale bar: 50 μm). (H) GSK3β with the proteins that are closely related to mitochondrial function and structure. (I) Graphical representation of molecular binding between GSK3β and TFAM protein obtained with the HDOCK server. (J) Western blot and quantitative analyses of TFAM and cGAS. (K, L) Co‐immunoprecipitation using TFAM antibody in LA post‐MI and in cells with or without H_2_O_2_ induction. *p < 0.05, **p < 0.01, ***p < 0.001, ****p < 0.0001. cGAS, cyclic GMP‐AMP synthase.

We next investigated whether there is a direct association between HDAC6 and GSK3β. We performed co‐immunoprecipitation using an HDAC6 antibody to specifically pull down GSK3β in the LA tissues of sham‐operated and post‐MI mice, compared to the IgG isotype control. Interestingly, we observed HDAC6 binding to GSK3β in the LA mitochondria of post‐MI mice (Figure 6A). In vitro experiments showed similar results. Using a GSK3β antibody, we specifically pulled down HDAC6 with or without H_2_O_2_ induction and observed that HDAC6 and GSK3β form a complex under H_2_O_2_ intervention (Figure 6B). To further explore this interaction, we used an HDAC6 antibody to pull down GSK3β; additional results were revealed. Without H_2_O_2_ intervention, HDAC6 showed little association with GSK3β or TOM20. However, under H_2_O_2_ stimulation, there was a significant association among these three proteins (Figure 6C). Immunofluorescence also showed that HDAC6 and TOM20 are co‐localized in the LA of post‐MI mice (Figure 6D). Based on these findings, we hypothesize that the HDAC6 and GSK3β complex may move into the mitochondria to work in it.

To test this hypothesis, we isolated mitochondrial proteins and cytosolic proteins. Under H_2_O_2_ induction, the levels of HDAC6 and GSK‐3β in mitochondria were increased compared to the conditions without H_2_O_2_. When the HDAC6 gene was silenced, H_2_O_2_ did not affect the mitochondrial levels of GSK3β. Conversely, overexpression of HDAC6 increased the level of GSK3β (Figure 6E,F). In the cytosol, we observed a similar trend in HDAC6 levels, whereas there were no significant changes in GSK3β levels among the four experimental groups (Figure 6E,F). The immunofluorescence evaluation of H_2_O_2_‐induced HL1 cells revealed increased colocalization of HDAC6 with GSK3β or TOM20 (Figure 6G). These results suggest that HDAC6 binds to the inactivated GSK3β within the intracellular place and partially translocates into the mitochondria under the induction of H_2_O_2_, thereby affecting mitochondrial function and ROS generation.

### 3.8 GSK3β Interacts With TFAM Within Mitochondria, Impairing TFAM Binding to mtDNA and Exacerbating mtDNA Damage

To further elucidate the relationships between GSK‐3β and mitochondria, we mapped GSK3β with proteins closely related to mitochondrial function and structure by using the STRING 12.0 database (https://string‐db.org/), and the interactions were visualized by Cytoscape 3.10 [33]. As shown in Figure 6H, 15 proteins were connected by a functional network, but only TFAM was related to GSK3β. Notably, most of the proteins were associated with TFAM, suggesting the central role of TFAM in maintaining mitochondrial structure and function. The HDOCK results predicted a potential interaction between GSK3β and TFAM (Figure 6I), with a binding free energy of the complex at −12.3 kcal/mol, a high docking score at −283.99, and 0.9358 as the confidence score. The possible binding amino acid sites include ARG‐156, GLN‐206, ARG‐232, and LYS‐86 (Figure 6I). The expression of TFAM was decreased in MI mice, but HDAC6 knockout reversed this change; the use of cyclic GMP‐AMP synthase (cGAS) resulted in the opposite trend (Figure 6J), suggesting that mtDNA is damaged and released into the cytoplasm. Immunoprecipitation using the TFAM antibody in vitro and in vivo confirmed that under oxidative stress, GSK3β forms a complex with TFAM, but no interaction with HDAC6 was detected, suggesting that GSK3β and HDAC6 dissociate in the mitochondria (Figure 6K,L). This finding was further supported by the result that HDAC6 silencing attenuated this interaction (Figure 6L). Taken together, these observations indicate that in mitochondria, after GSK3β dissociates from HDAC6, it binds to TFAM, competitively interfering with the binding of TFAM to mtDNA, thereby impairing the transcription and stability of mtDNA, which in turn affects mitochondrial function.

### 3.9 HDAC6 Overexpression Activated the Wnt3a‐GSK3β Signaling Pathway, Promoting Oxidative Stress and Pyroptosis

**FIGURE 7:**
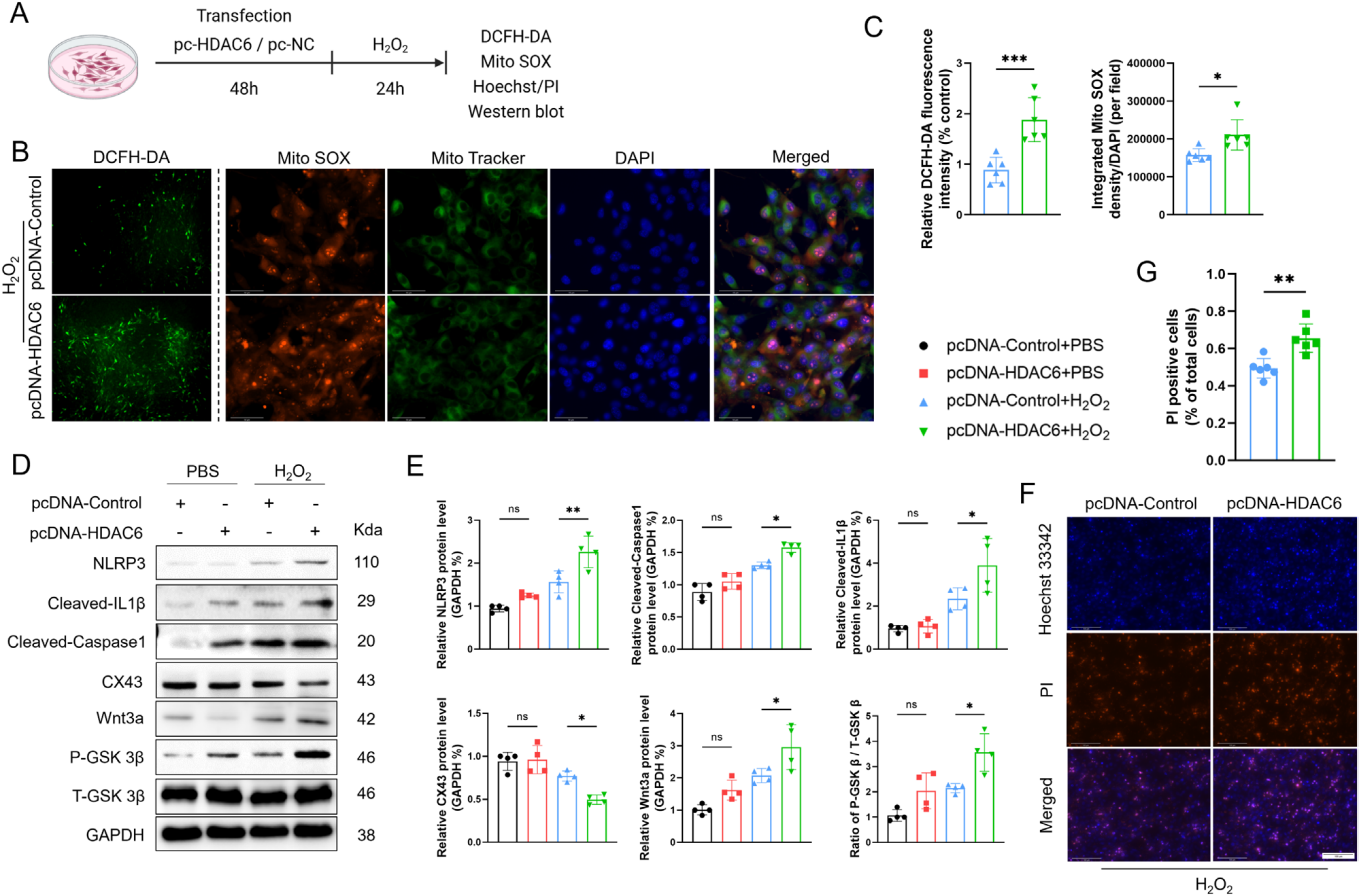
HDAC6 overexpression activates the Wnt3a/GSK3β signaling pathway, promoting oxidative stress and pyroptosis. (A) The transfection and treatment experimental protocol. (B, C) DCFH‐DA (scale bar: 100 μm) and Mito SOX (red)/Mito Tracker (green) (scale bar: 50 μm) staining of cells after pcDNA transfection and H_2_O_2_ induction. The results of the quantitative analysis of fluorescence intensity ae shown. (D, E) Western blot and quantitative analysis of NLRP3, cleaved‐caspase1, cleaved‐IL1β, CX43, Wnt3a, β‐catenin, P‐GSK3β, and T‐GSK3β in cells with HDAC6 overexpression. (F, G) Hoechst/PI staining of cells in each group. The results of the quantitative analysis of PI‐positive cells. *p < 0.05, **p < 0.01, ***p < 0.001, ****p < 0.0001.

We next evaluated the impact of HDAC6 overexpression on oxidative stress, pyroptosis, CX43 expression, and the Wnt3a‐GSK3β signaling pathway in vitro. We transfected cells with either pcDNA‐HDAC6 or pcDNA‐Control, followed by H_2_O_2_ induction for 24 h (Figure 7A). We divided HL1 cells into four groups: pcDNA‐Control+PBS, pcDNA‐HDAC6 + PBS, pcDNA‐Control+H_2_O_2_, and pcDNA‐HDAC6 + H_2_O_2_. Compared to the control group, HDAC6 overexpression was associated with elevated levels of DCFH‐DA and MitoSOX staining signals, indicating enhanced mitochondrial oxidative stress production (Figure 7B,C). The use of pcDNA‐HDAC6 exacerbated the reduction of CX43 and increased the expressions of Wnt3a, p‐GSK3β, and pyroptosis‐related proteins, that is, NLRP3, cleaved‐caspase1, and cleaved‐IL1β (Figure 7D,E). Likewise, the quantitative Hoechst/PI staining data revealed that HDAC6 overexpression also increased the number of PI^+^ cells compared to the control treatment (Figure 7F,G). These results thus indicate that in HL1 cells, HDAC6 overexpression can increase mitochondrial oxidative stress production and pyroptosis via modulations of the Wnt3a‐GSK3β signaling pathway.

### 3.10 GSK3β Acted as a Key Player in the HDAC6‐Mediated Wnt3a Signaling Activation in the HL1 Cells' Response to H_2_O_2_

**FIGURE 8:**
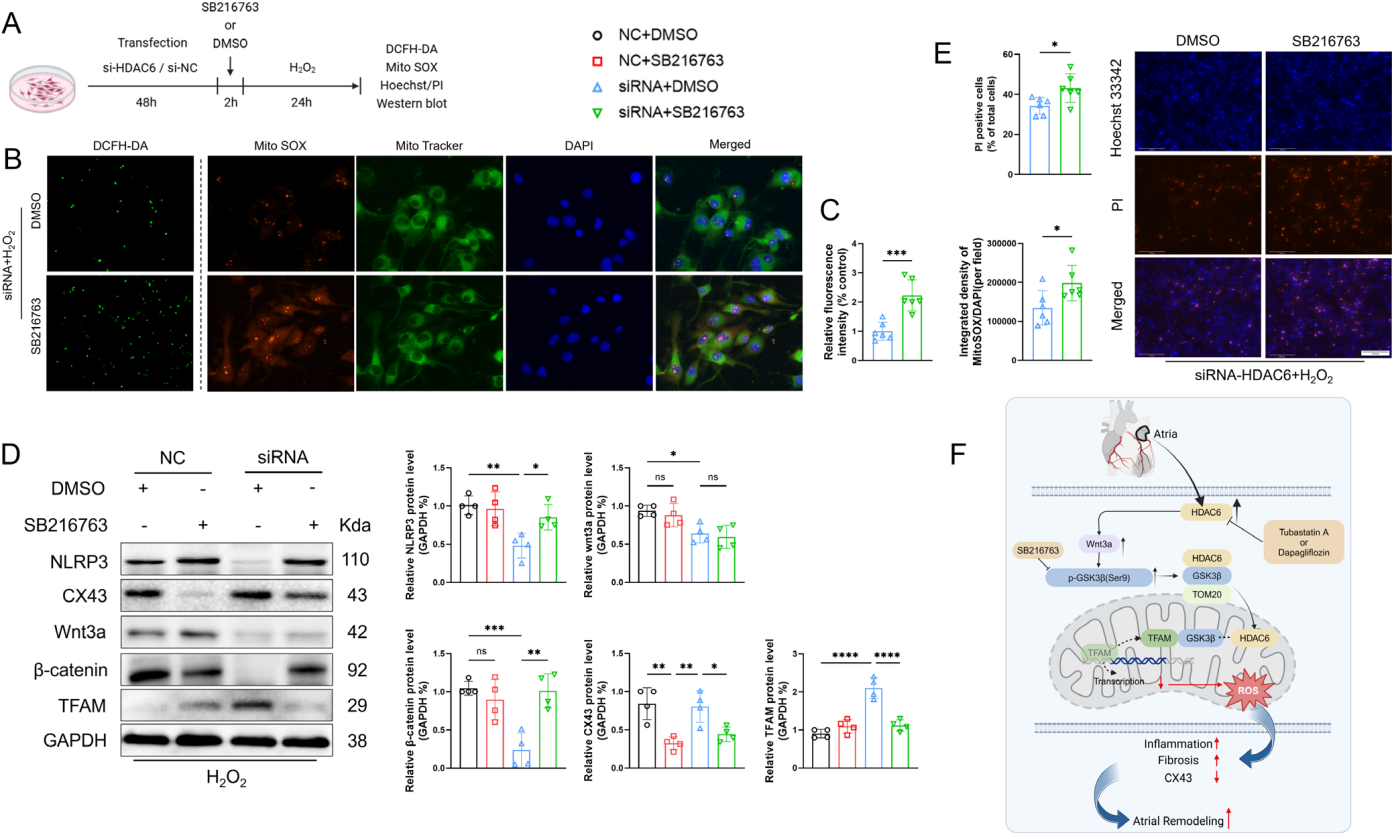
HDAC6 promotes oxidative stress by modulating the Wnt/GSK3β signaling pathway. (A) The transfection and treatment experimental protocol. (B, C) DCFH‐DA (scale bar: 100 μm) and Mito SOX (*red*)/Mito Tracker (*green*) (scale bar: 50 μm) staining of cells after siRNA transfection, SB216763 treatment, and H_2_O_2_ induction. The results of the quantitative analysis of fluorescence intensity are presented. (D) Western blot and quantitative analysis of wnt3a, β‐catenin, NLRP3, and CX43 in cells in each group. (E) Hoechst/PI staining of cells after transfection and treatment. The results of the quantitative analysis of PI‐positive cells. (F) Schematic illustration of the role of HDAC6 in regulating atrial remodeling post‐MI. *p < 0.05, **p < 0.01, ***p < 0.001, ****p < 0.0001. OCR, oxygen consumption rate.

Finally, to further test our hypothesis that GSK3β may play a role in this process, we applied the GSK3β inhibitor SB216763 to the culturing condition of HL1 cells that were transfected with siRNA‐HDAC6 or nontargeted control siRNA for 24 h (Figure 8A) and then subjected to the biological analyses. Compared to the siRNA‐HDAC6 + H_2_O_2_ group, SB216763 loading diminished the HDAC6 silencing‐induced beneficial changes, including not only the reductions of the mitochondrial oxidative stress production parameters (DCFH‐DA‐fluorescence intensity and MitoSOX/DAPI ratio) and the number of PI^+^ cells but also the reductions of the pyroptosis‐related protein NLRP3 levels and the enhancement of the CX43 protein level (Figure 8B–E). Moreover, the level of Wnt3a was not affected by the inhibitor. All of the above suggest that HDAC6 may modulate inflammation, mitochondrial oxidative stress production, and pyroptosis via the GSK3β‐mediated Wnt3a signaling pathway.

## 4 Discussion

In this study we focused on the potential role(s) of HDAC6 in MI‐related AR in mice. The study's most significant finding is that HDAC6 deficiency was resistant to MI‐induced atrial mitochondrial damage, cardiomyocyte hypertrophy, fibrosis, and enlargement in mice. At the molecular and cellular levels, HDAC6 deficiency prevented (i) the elevations of plasma inflammatory cytokines (IL‐18 and IL‐1β) and LA tissue chemokines (OPN), (ii) pyroptosis‐related molecules (NLRP3, cleaved‐caspase1, and cleaved‐IL‐1β), (iii) mitochondrial biogenesis (PGC‐1α and TFAM) and oxidative stress production (Mito SOX and DCHF‐DA), (iv) fibrosis (collagen type I and α‐SMC)‐ and gap‐junction (CX43)‐related proteins, (v) Wnt3a, p‐GSK3β, and β‐catenin signal proteins, and (vi) galectin3^+^ and PI^+^ cells. An HDAC6 inhibitor mimicked HDAC6‐mediated atrial protection in post‐MI mice. We also observed that the efficacy of the HDAC6 inhibitor TubA is comparable to that of a SGLT2 inhibitor, that is, Dap, for the inhibition of HDAC6 signaling in HDAC6^+/+^ mice. In vitro, intracellular GSK3β binds to HDAC6 and translocates into the mitochondria. Following disassociation with HDAC6, GSK3β competitively binds with TFAM to mtDNA. The silencing (and the overexpression) of HDAC6 respectively decreased (and increased) mitochondrial oxidative stress production and pyroptosis, accompanied by negative (and positive) modulations of the Wnt3a/GSK3β signaling pathway in HL1 cells under oxidative stress conditions, providing evidence of a potential mechanistic explanation of an HDAC6‐mediated Wnt3a/GSK3β signaling pathway in the modulation of the atrial fibrosis and remodeling in post‐MI mice under our experimental conditions. The mechanisms underlying the amelioration of HDAC6 inhibition‐mediated atrial protective actions in post‐MI mice are schematically represented in Figure 8F.

The occurrence of atrial arrhythmia after MI can be influenced by various factors, with AR playing the central role. AR refers to a range of pathophysiological changes in the structure, mechanical function, and electric, ionic, and molecular environment of the LA [34]. The remodeling of the LA extracellular matrix can increase the vulnerability to atrial arrhythmia after MI [35]. Increasing evidence suggests a strong connection between AR and oxidative stress, as well as inflammation [36]. After an MI, the atria are exposed to significant oxidative stress, combined with the potential development of heart failure; the atria exhibit enlargement, fibrosis, ROS generation, and infiltration of inflammatory cells [37] which is consistent with our present findings.

HDAC6, a class IIb member of the HDAC family, is located primarily in the cytoplasm and participates in various cellular processes including cell migration, stress response, and signal transduction [15]. It has been shown that HDAC6 is linked to cellular oxidative stress, but it remains unclear whether HDAC6 inhibition has a protective effect on atrial cardiomyocytes after MI. LA enlargement and fibrosis are hallmarks of arrhythmogenic structural remodeling, and they are closely associated with atrial arrhythmias [38, 39]. Both of these changes can be promoted after MI and reversed by HDAC6 knockout. CX43, a member of the family of gap junction proteins, can lead to anisotropic conduction and prolonged conduction time when reduced. This can increase susceptibility to atrial fibrillation [40]. Changes in atrial electrical conduction can be partially observed through the P‐wave duration and PR interval in ECG. HDAC6 deletion maintains the level of CX43 protein and attenuates MI‐induced changes in both P‐wave duration and PR interval, indicating that HDAC6^−/−^ may prevent atrial structural and electrophysiological remodeling post‐MI.

We observed mitochondrial dysfunction and ROS generation in the murine LA post‐MI. Both of these factors are involved in the activation of NLRP3 inflammasome assembly, subsequently activating caspase‐1 and IL‐1β, which can induce pyroptosis and AR [17]. Galectin‐3, a member of the galectin family secreted by macrophages, engages in fibrosis and inflammation, contributing to post‐MI AR [41]. Our present experiments revealed that after the knockout of HDAC6, the activation of NLRP3‐related proteins and the secretion of galectin‐3 were reduced, significantly alleviating atrial inflammation. This is likely due to the restoration of mitochondrial function and structure.

The Wnt/GSK3β signaling pathway is closely associated with oxidative stress and lung fibrosis [42]. The current research on HDAC6 and Wnt3a in AR is limited. Our present results demonstrated that the level of Wnt3a is increased in the LA post‐MI. HDAC6 deletion diminished this change, whereas the overexpression of HDAC6 produced the opposite effect. GSK3β, as a serine/threonine protein kinase, is one of the main regulators of energy metabolism in cells. When the Wnt pathway is inactivated, Axin and APC function as scaffold proteins, forming a complex with GSK3β and β‐catenin. GSK3β phosphorylates β‐catenin, leading to its subsequent ubiquitination and degradation [43]. Upon Wnt activation, the complex dissociates, and GSK3β is phosphorylated at the Ser9 site, thereby losing its activity. Consequently, β‐catenin is no longer phosphorylated and degraded, resulting in its accumulation in the cytoplasm and translocation to the nucleus, thereby promoting the transcription of profibrotic genes [44] which is consistent with the fibrotic changes we observed in this study. After the inhibition of GSK3β activity by SB216763, the impact of HDAC6 silencing on Wnt3a remains unchanged, but its protective effect on ROS generation and pyroptosis is diminished. This suggests that the decrease in ROS generation and inflammation resulting from HDAC6 deletion is mediated by the reduction of Wnt/GSK3β signaling activation.

As the primary organelles responsible for intracellular ROS production, mitochondria can lead to arrhythmias when their function is disordered [45]. In our present investigation, the number, biosynthetic ability, cristae morphology, and membrane integrity of mitochondria in the mouse LA were all impaired by MI. The knockdown of HDAC6 reversed these changes, which is consistent with the findings of an earlier transcriptomic study [19]. The expanded sarcoplasmic reticulum may be a compensatory response to mitochondrial dysfunction. The relationship between HDAC6 and mitochondria seems to be multifaceted. In myocardial ischemia/reperfusion injury in diabetic mice, the inhibition of HDAC6 can reduce myocardial mitochondrial fission by decreasing the level of tumor necrosis factor alpha and recover mitochondrial complex I [46]. In our present study, HDAC6 bound with TOM20 and GSK3β under oxidative stress conditions in atrial myocytes. TOM20 is a protein on the mitochondrial outer membrane, and it participates in the import of cytosolic proteins into mitochondria [47]. Interestingly, HDAC6 is also extensively involved in the intracellular transport process. We observed that after H_2_O_2_ stimulation, the mitochondrial content of GSK3β increased, and this change was abolished by HDAC6 silencing and enhanced by its overexpression, indicating that HDAC6 is related to the translocation of GSK3β into mitochondria. This is likely one of the reasons leading to the alterations in mitochondrial cristae and membrane structure, which can result in mitochondrial electron transport chain dysfunction, thereby increasing the production of ROS [48]. The role of GSK3β in oxidative stress remains controversial. It has been suggested that the activation of GSK3β can enhance ischemia/reperfusion‐induced cardiac damage by affecting the Nrf2/Nqo1 signaling axis [49]. However, it was also demonstrated that the loss of GSK3β activity reduces the phosphorylation of the 6b subunit of the electron transport chain, leading to defects in mitochondrial complex IV [50]. Inhibition of GSK3β also significantly impacts mitochondrial function, increasing the production of superoxide [51]. These varying findings may be due to GSK3β's extensive involvement in energy metabolism processes. A certain level of GSK3β activity is necessary, as both its overexpression and excessive inhibition can affect mitochondrial structure and ROS production. Additionally, its role may differ across various tissues and experimental conditions. In mitochondria, TFAM acts as a transcription factor that can bind to multiple sites on mtDNA, including the heavy strand promoter, light strand promoter, and the D‐loop region, regulating its transcription and replication to maintain mtDNA stability [52]. We observed that after GSK3β translocates into mitochondria, it binds to TFAM, which affects the interaction between TFAM and mtDNA, thereby downregulating the transcription and stability of mtDNA. HDAC6 deletion reduces the transport of GSK3β to mitochondria and maintains the normal function of mtDNA. cGAS, a cytoplasmic DNA sensor, can directly bind to mtDNA. In the present experiments, we detected elevated levels of cGAS in the atria of MI mice, indicating the leakage of mtDNA to cytoplasm, and this elevation was reversed following HDAC6 deletion. Overall, HDAC6 appears to increase intracellular oxidative stress by affecting the homeostasis and superoxide production of mitochondria in LA post‐MI through the translocation of GSK3β from the cytoplasm into the mitochondria, and this competitively affects TFAM's binding to mtDNA (Figure 8F).

Given the role of HDAC6^−/−^ in post‐MI AR, we further investigated the effects of pharmacologic inhibition of this target. TubA is the most commonly used specific inhibitor of HDAC6. Dapagliflozin is an SGLT2 inhibitor that is commonly used clinically to treat heart failure. A 2022 study indicated that SGLT2 inhibitors have a strong affinity with HDAC6, [31] which suggests that they may act as a targeted inhibitors of HDAC6. We tested this hypothesis by conducting in vivo experiments, and the results confirmed that Dap does exhibit HDAC6‐inhibitory efficacy that is comparable to HDAC6 inhibitors. Further experiments revealed that inhibiting HDAC6 with these two drugs can attenuate AR after MI in mice and reduce the level of ROS in the mouse atria, thus demonstrating the therapeutic potential of HDAC6. While Dap may improve AR by alleviating post‐MI heart failure, our findings suggest that its protective effects are at least partly attributable to direct inhibition of HDAC6.

In summary, our experiments revealed that the expressions of the HDAC6 gene and protein were increased in the left atrial tissues of mice subjected to ischemic surgery. HDAC6 deletion alleviated MI‐induced AR via the reduction of atrial inflammation, mitochondrial biogenesis, damage, oxidative stress production, and cardiomyocyte pyroptosis that was mediated by Wnt3a/GSK3β signaling inactivation in mice under our experimental conditions. The pharmacological inhibition of HDAC6 mimics the atrialoprotective effects of genetic HDAC6 deletion. This study is apparently the first to report the beneficial action of HDAC6 inhibition on remodeling remote from the infarct area, suggesting that a selective HDAC6 inhibitor might have potential utility in the management of atrial fibrosis and remodeling in patients with MI.

## 5 Limitation

Although our study demonstrates the role of HDAC6 in the atria post‐MI, several issues remain unsolved. First, we could not perform burst pacing of the LA to induce AF in mice or conduct optical mapping of the atria. As a result, we do not have detailed descriptions of the specific electrophysiological changes or the incidence of AF. Second, we also could not provide direct evidence showing that Dap inhibits AR through inhibiting HDAC6 in post‐MI mice. Third, we could not create atrial cardiomyocyte‐specific deletion and overexpression of HDAC6 mice to explore the role of HDAC6 in MI‐induced AR and arrhythmia in mice. Further studies are necessary to investigate these issues.

## Author Contributions

S.S. conceived the project, designed and performed experiments, implemented and analyzed the data, interpreted data, prepared figures, and drafted and wrote the article. J.F., L.Z., J.H., M.Z., C.H. participated in experiments and performed the blinded data analysis of immunostaining. S.L. discussed the results and revised and critically reviewed the article. X.W.C. conceived the project, designed and performed experiments, interpreted data, and revised and critically reviewed the article. All authors read and approved the final version of the article.

## Ethics Statement

All procedures of mouse experiments were approved by the Institutional Animal Care and Use Committee at Yanbian University.

## Conflicts of Interest

The authors declare no conflicts of interest.

## Supporting information


Data S1.

## Data Availability

The data that support the findings of this study are available from the corresponding author upon reasonable request. Source data are available online.
