# Supplementary material for: Histone Deacetylase 6 Controls Atrial Fibrosis and Remodeling in Postinfarction Mice Through the Modulation of Wnt3a/GSK‐3β Signaling
Source: FASEB J. 2025 May 16;39(10):e70650. doi: 10.1096/fj.202500371R (PMC12083057; doi:10.1096/fj.202500371R)
Supplement: Supplementary file 1 — Data S1. [file FSB2-39-e70650-s001.docx]

**Supplementary Data**

**Histone Deacetylase 6 Controls Atrial Fibrosis and Remodeling in Post-Infarction Mice Through the Modulation of Wnt3/GSK-3β Signaling**

Shangzhi Shu, MD; Junqiao, Fang, MD; Longguo Zhao, MD; Jiatong Han, MD; Meiping Zhang, MD; Chaoqun Huang, MD; Xian Wu Cheng, MD, PhD, FAHA; Shuyan Li, MD, PhD

**Supplementary Figure S1-S4 and Figure legends and Supplementary Table S1**

Correspondence:

Shuyan Li, MD, Department of Cardiology, First Hospital of Jilin University, No. 71 Xinmin Street, Changchun, Jilin PR 130021, China

Email: li_sy@jlu.edu.cn

Xianwu Cheng, MD, Department of Cardiology and Hypertension, Yanbian University Hospital, 1327 Juzijie, Yanji, Jilin PR 133000, China.

Email: chengxw0908@163.com

**Supplementary Data**


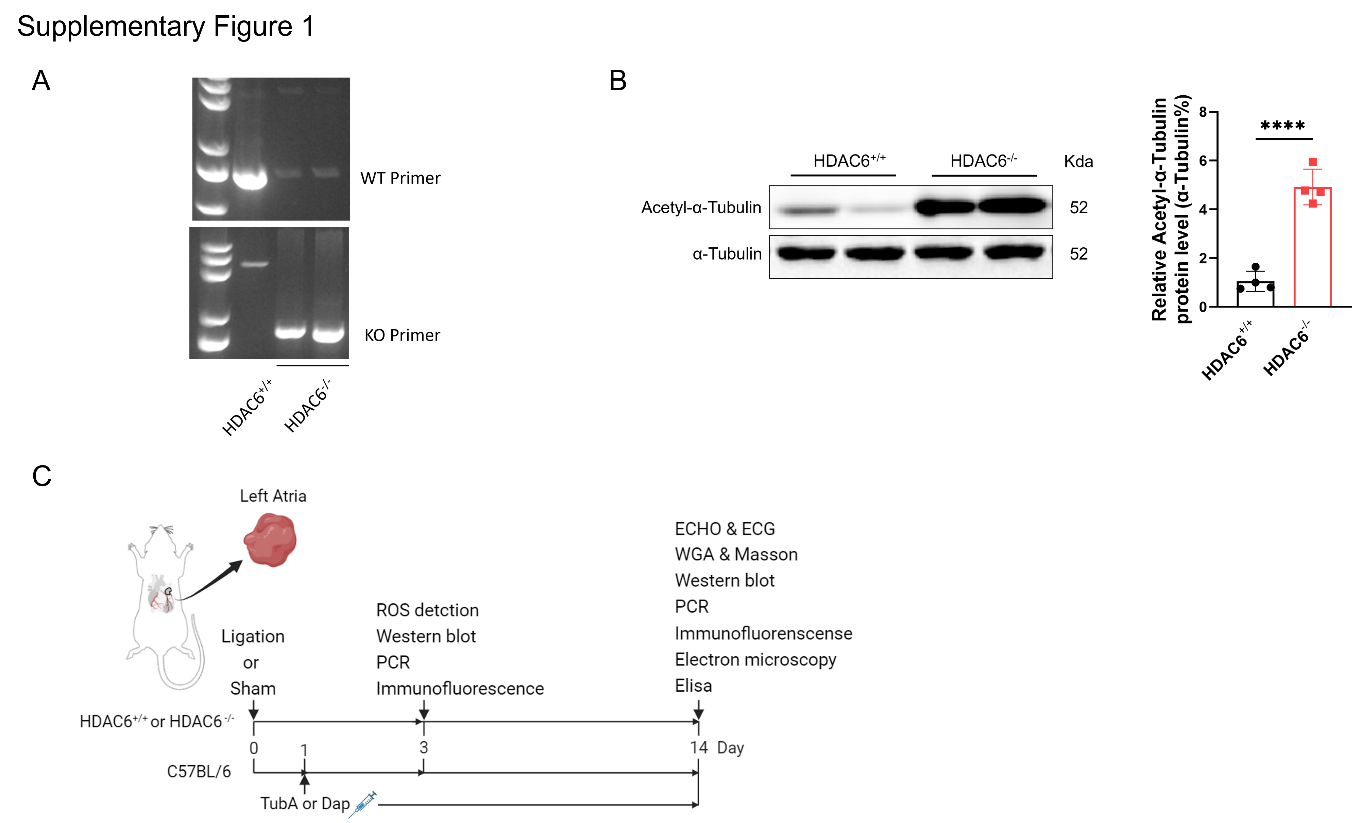


**Supplementary Figure 1.** **A**: Agarose gel electrophoresis of HDAC6 knockout mice and C57BL/6 mice. **B**: Western blot of acetyl-α-tubulin protein. **C**: The in vivo experimental protocols. The six group of mice: HDAC6^+/+^-Sham, HDAC6^−/−^-Sham, HDAC6^+/+^-MI, HDAC6^−/−^-MI, MI-TubA, and MI-Dap. The experimental protocols shown in the figure were conducted on the 3rd and 14th days after MI surgery, respectively. MI: myocardial infarction, ROS: reactive oxygen species, Dap: dapagliflozin, TubA: tubastatin A.


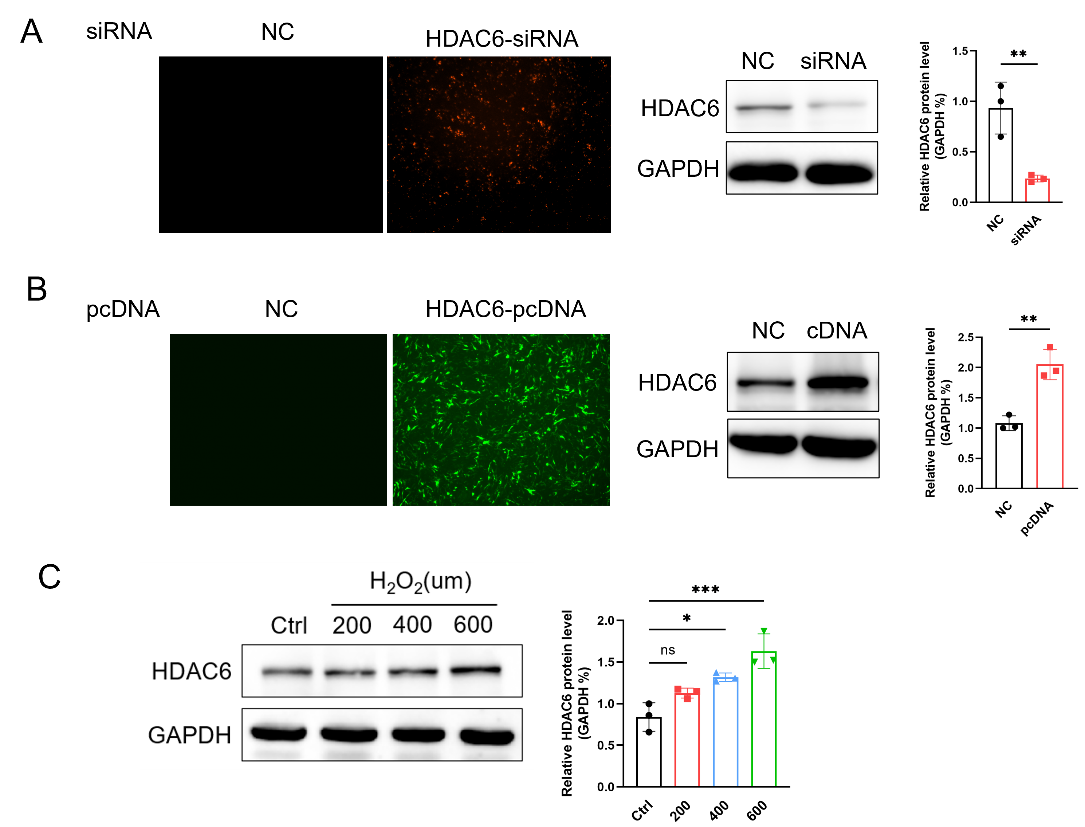


**Supplementary Figure 2.** **A:** The transfection efficiency of HL1 cells with siRNA-HDAC6. The plasmid carries red fluorescence. A western blot of HDAC6 in transfected cells is shown. **B:** The transfection efficiency of HL1 cells with pcDNA-HDAC6. The plasmid carries green fluorescence. A western blot of HDAC6 in transfected cells is shown. **C:** The expression of HDAC6 in HL1 cells under stimulation with different concentrations of H_2_O_2_ as shown by western blotting.


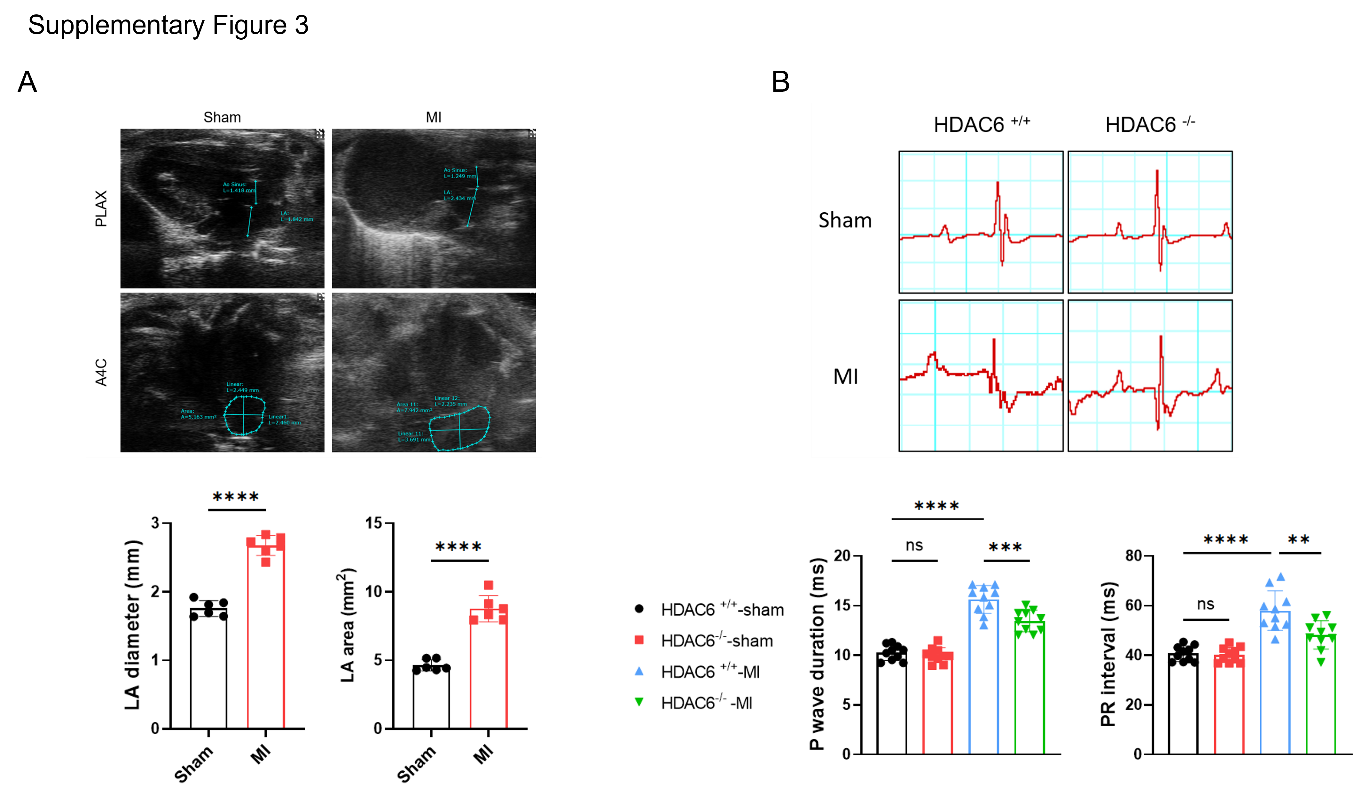


**Supplementary Figure 3.** **A:** Echocardiograms of sham-operated and MI mice. The results of the quantitative analysis of the LA diameter and LA area are shown. **B:** Electrocardiograms of sham and MI mice. The quantitative analysis results regarding the P-wave duration and PR interval are shown. *p<0.05, **p<0.01, ***p<0.001, ****p<0.0001. LA: left atrium.


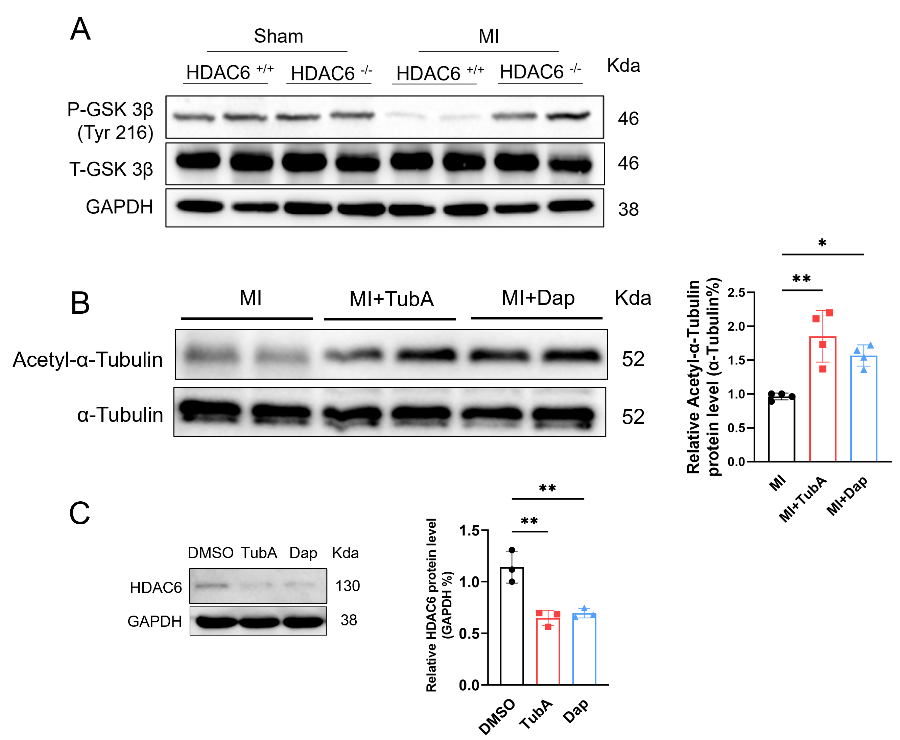


**Supplementary Figure 4.** **A:** Western blot of P-GSK3β at Tyr 216 site and T GSK3β. **B:** Western blot and quantitative analysis of acetyl-α-tubulin of MI, MI+TubA and MI+Dap mice. **C:** Western blot and quantitative analysis of HL-1 cells treated with TubA or Dap. Both drugs were administered at a concentration of 5 μM for 12 hours. The DMSO group served as the negative control. *p<0.05, **p<0.01, ***p<0.001, ****p<0.0001.

| mRNA | Forward primer | Reverse primer |
| --- | --- | --- |
| HDAC6 | TCCACCGGCCAAGATTCTTC | CAGCACACTTCTTTCCACCAC |
| Wnt3a | CTCCTCTCGGATACCTCTTAGTG | GCATGATCTCCACGTAGTTCCTG |
| β-catenin | ATGGAGCCGGACAGAAAAGC | CTTGCCACTCAGGGAAGGA |
| PGC1α | TATGGAGTGACATAGAGTGTGCT | CCACTTCAATCCACCCAGAAAG |
| TFAM | ATTCCGAAGTGTTTTTCCAGCA | TCTGAAAGTTTTGCATCTGGGT |
| NLRP3 | ATTACCCGCCCGAGAAAGG | TCGCAGCAAAGATCCACACAG |
| CASP1 | ACAAGGCACGGGACCTATG | TCCCAGTCAGTCCTGGAAATG |
| IL1β | GCAACTGTTCCTGAACTCAACT | ATCTTTTGGGGTCCGTCAACT |
| OPN | ATCTCACCATTCGGATGAGTCT | TGTAGGGACGATTGGAGTGAAA |
| OPA1 | TGGAAAATGGTTCGAGAGTCAG | CATTCCGTCTCTAGGTTAAAGCG |

**Supplementary Table 1.** Sequences of PCR primer.
